# An Immunogenicity Report for the Comparison between Heterologous and Homologous Prime-Boost Schedules with ChAdOx1-S and BNT162b2 Vaccines

**DOI:** 10.3390/jcm10173817

**Published:** 2021-08-25

**Authors:** Alexandre Vallée, Marc Vasse, Laurence Mazaux, Brigitte Bonan, Carline Amiel, Sara Zia-Chahabi, Aurélie Chan-Hew-Wai, Eric Farfour, Eve Camps, Pauline Touche, Flavie Barret, François Parquin, David Zucman, Erwan Fourn

**Affiliations:** 1 Department of Clinical Research and Innovation, Foch Hospital, 92150 Suresnes, France; p.touche@hopital-foch.com (P.T.); f.barret@hopital-foch.com (F.B.); 2 Biology Department, Foch Hospital, 92150 Suresnes, France; m.vasse@hopital-foch.com (M.V.); l.mazaux@hopital-foch.com (L.M.); s.zia-chahabi@hopital-foch.com (S.Z.-C.); e.farfour@hopital-foch.com (E.F.); 3 INSERM, UMR-S1176, Université Paris-Saclay, 94000 Le Kremlin-Bicêtre, France; 4 Hospital Pharmacy, Foch Hospital, 92150 Suresnes, France; b.bonan@hopital-foch.com (B.B.); a.chan-hew-wai@hopital-foch.com (A.C.-H.-W.); e.camps@hopital-foch.com (E.C.); 5 Service de Médecine du Travail, 92150 Suresnes, France; c.amiel-taieb@hopital-foch.com; 6 Thoracic Intensive Care Unit, Foch Hospital, 92150 Suresnes, France; f.parquin@hopital-foch.com; 7 Réseau Ville-Hôpital, Service de Médecine Interne, Foch Hospital, 92150 Suresnes, France; d.zucman@hopital-foch.com (D.Z.); e.fourn@hopital-foch.com (E.F.)

**Keywords:** COVID-19, COVID-19 vaccine, ChAdOx1-S, BNT162b2, immunogenicity

## Abstract

Background: There is a small amount of immunological data on COVID-19 heterologous vaccination schedules in humans. We assessed the immunogenicity of BNT162b2 (Pfizer/BioNTech) administered as a second dose in healthcare workers primed with ChAdOx1-S (Vaxzevria, AstraZeneca). Methods: 197 healthcare workers were included in a monocentric observational study in Foch hospital, France, between June and July 2021. The main outcome was the immunogenicity measured by serum SARS-CoV-2 IgG antibodies. Results: 130 participants received the ChAdOx1-S/BNT vaccine and 67 received the BNT/BNT vaccine. The geometric mean of IgG antibodies was significantly higher in the BNT/BNT vaccine group compared to the ChAdOx1-S/BNT vaccine group, namely 10,734.9, 95% CI (9141.1–12,589.3) vs. 7268.6, 95% CI (6501.3–8128.3), respectively (*p* < 0.001). However, after adjustment for time duration between the prime and second vaccinations, no significant difference was observed (*p* = 0.181). A negative correlation between antibody levels and time duration between second dose and serology test was observed for the BNT/BNT vaccine (*p* < 0.001), which remained significant after adjustment for all covariates (*p* < 0.001), but not for the ChAdOx1-S/BNT vaccine (*p* = 0.467). Conclusions: Heterologous and homologous schedules of ChAdOx1-S and BNT vaccines present robust immune responses after the second vaccination. The results observed were equivalent after adjustment for covariates and emphasize the importance of flexibility in deploying mRNA and viral vectored vaccines. Nevertheless, applying the ChAdOx1-S schedule vaccination for the heterologous second dose of BNT was associated with decreased IgG antibody levels compared to the homologous BNT/BNT vaccination.

## 1. Introduction

As of June 2021, SARS-CoV-2 infection has caused more than 185 million infections worldwide with a total death toll of more than 4.0 million. The COVID-19 pandemic has impacted the world in economic, social and health terms. Herd immunity remains the fundamental way to reduce the burden of the viral pandemic [1]. A massive vaccine campaign has been started in several countries with different vaccines (Moderna (mRNA-1273), Pfizer/BionTech (BNT162b2), Sputnik V, AstraZeneca (ChAdOx1-S)). Epidemiologic studies have observed that COVID-19 vaccines should reduce the rates of infection, which will eventually yield to herd immunity when around 70% of the populations become fully vaccinated [2]. Nevertheless, on 15 March 2021, numerous European countries stopped ChAdOx1-S vaccine use as a precaution to investigate the death of a few dozen patients developing blood clots associated with deep vein thrombosis (DVT) [3]. In Europe, on 15 March 2021, only 30 suspect cases of DVT had been observed [4]. However, on 22 March 2021, the ChAdOx1-S vaccine campaign resumed in many countries, including France [5].

On 19 March 2021, the French High Authority for Health (HAS) announced that it was recommending the ChAdOx1-S vaccine only for people aged over 55 years. This decision was taken based on the rare cases of DVT occuring only in people aged under 55 years. The European Medicine Agency (EMA) asked not to ignore rare events, namely serious incidents that occurred among the 20 million vaccinations in Europe and the United Kingdom, which are 18 occurrences of cerebral venous thrombosis and seven disseminated intravascular coagulations [4].

In France, these changes in vaccine strategy induced as alternative the possibility of sequentially administering different COVID-19 vaccines, known as heterologous schedules. Thus, the French government advised administering a second dose with an mRNA (BNT or Moderna) vaccine in people primed with the ChAdOx1-S vaccine, even without supporting data regarding the immunogenicity of this schedule.

Heterologous strategies were not novel as they have been used in multiple HIV vaccines [6], Ebola vaccines [7] and in influenza vaccines [8]. However, few efficacy data using heterologous schedules incorporating COVID-19 vaccine are available in the world [9]. Previous studies have shown that a second vaccination with BNT was associated with increased anti-spike IgG levels for ChAdOx1-S-primed peopled compared to those having only one ChAdOx1-S dose [10,11,12]. However, contradictory anti-spike IgG levels were observed between participants who received homologous BNT/BNT vaccines or heterologous ChAdOx1-S/BNT vaccines with similar rates [10,13] and higher rates for heterologous ChAdOx1-S/BNT vaccines [9,14,15]. Nevertheless, no evidence of immune response outcomes with heterologous vaccine strategies is clearly available to date for the COVID-19 pandemic [16].

Thus, to answer this fundamental question, we designed in the Foch hospital, Suresnes, France, the retrospective ASTERMIX Foch COVID-19 study, in real-life practice according to the French recommendations, to evaluate the immune responses to heterologous schedules deploying ChAdOx1-S/BNT vaccines to the homologous BNT/BNT vaccines.

## 2. Methods Design

The ASTERMIX Foch COVID-19 study is a retrospective, cross-sectional and monocenter study. Participants were healthcare workers, adults (aged over 18 years) who had no previous COVID-19 infection. In France, the prime dose with ChAdOx1-S vaccine was not recommended for people younger than 55 years for vaccination since 19 March 2021. In our study, participants aged over 55 years were excluded from the study due to, after prime ChAdOx1-S vaccination, second BNT vaccination was not being recommended for people over 55 years. Exclusion criteria were the presence of clinically significant acute illness or temperature over 38 °C, clinical manifestations compatible with COVID-19 and any condition contraindicating or discouraging BNT administration for a second dose, including pregnancy, according to the French recommendations in March 2021.

The study was approved by the Foch IRB: IRB00012437 (approval number: 21-06-03) on 4 June 2021. A non-opposed consent was obtained from all participants.

### 2.1. Procedures

Healthcare workers received online and/or telephone screening to be invited for a serology test between June and July 2021. Two COVID-19 vaccines were used in our study. ChAdOx1-S is a replication-deficient chimpanzee adenovirus vectored vaccine, expressing the SARS-CoV-2 spike surface glycoprotein with a leading tissue plasminogen activator signal sequence. Administration was via 0.5 mL intramuscular injection into the upper arm. BNT is a lipid nanoparticle-formulated, nucleoside-modified mRNA vaccine encoding a trimerized SARS-CoV-2 spike glycoprotein. Administration is via 0.3 mL intramuscular injection into the upper arm.

Participants of the study either received two vaccinations for BNT four weeks apart or an initial dose of ChAdOx1-S followed by a heterologous boost with BNT 12 weeks later (on the scheme of homologous vaccination with a second dose of ChAdOx1-S), in accordance with the French recommendations on COVID-19 vaccines [17]. Vaccines were administrated by the occupational medical team and vaccination unit of the Foch hospital, Suresnes, France.

### 2.2. Covariates

Age and gender of healthcare workers, date of prime and second vaccination and date of serology test were reported. Different time durations were calculated as “time between second vaccination and serology test”, “time between prime and second vaccination” and “time between prime vaccination and serology test”. IgG antibody levels were reported for each participant between 30 and 60 days after the second vaccination.

### 2.3. Outcomes

The primary outcome is the serum SARS-CoV-2 IgG antibody level 30 to 60 days after the second vaccination.

### 2.4. Laboratory Method

SARS-CoV-2 IgG II Quant assays were performed on the Abbott Alinity i platform in accordance with the manufacturer’s package insert [18,19]. In this antibody CMIA test, the SARS-CoV-2 antigen-coated paramagnetic microparticles bind to the IgG antibodies that attach to the virus’s spike protein in the serum sample. The resulting chemiluminescence in relative light units (RLU) following the addition of anti-human IgG (mouse, monoclonal) acridinium labeled conjugate in comparison with the IgG II calibrator/standard indicates the strength of response, which reflects the quantity of IgG_SP_ present.

### 2.5. Statistical Analysis

Data for antibodies were presented as the geometric mean and 95% confidence interval (95% CI), as median and interquartile ranges for continuous variables and as number and percentage for categorical variables. Qualitative variables were compared using Fisher’s exact test, while a T-test or Mann–Whitney’s test was used for continuous variables. Linear correlations were performed for the relationship between each vaccine group and all covariates. Significance was defined by a *p* value  < 0.05. For each model, multivariate analyses were performed with adjustment for covariates (age, gender, and the different time durations reported between vaccination and serology test). Statistical analyses were performed using SAS software (version 9.4; SAS Institute, Carry, NC, USA).

## 3. Results

Between June and July 2021, 197 participants were tested in Foch hospital, Suresnes, France. Demographic characteristics are shown in Table 1. A total of 130 participants received the ChAdOx1-S/BNT vaccination and 67 received the BNT/BNT vaccination.

A significant difference was observed between the two groups for age (respectively, median of 37 (13) vs. 32 (11), *p* < 0.001) but not for gender (respectively, female, 104 (80.0%) vs. 59 (88.1%), *p* = 0.156).

**Figure 1:**
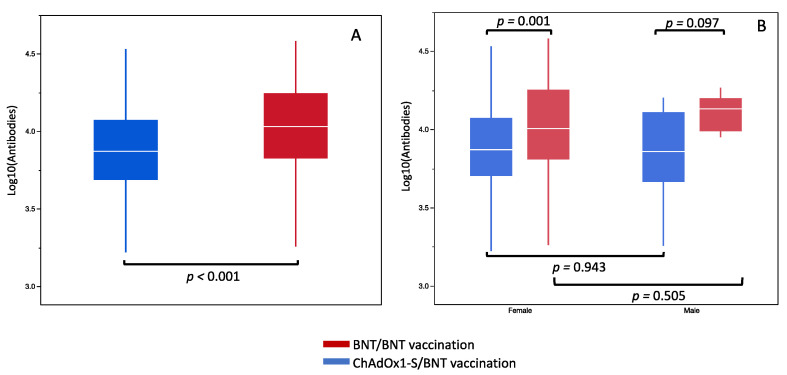
IgG antibody levels in overall population according to BNT/BNT and ChAdOx1-S/BNT (**A**) and according to gender (**B**).

In univariate analysis, the geometric mean of antibodies was significantly higher in the BNT/BNT vaccination group compared to the ChAdOx1-S/BNT vaccination group (respectively, 10,734.9, 95% CI (9141.1–12,589.3) vs. 7268.6, 95% CI (6501.3–8128.3) *p* < 0.001). The inclusion period was comprised between 30 and 60 days after the second dose, but a significant difference was observed among the two groups (respectively, 38 (7) days vs. 42 (9) days, *p* < 0.001). As expected, time duration between the first and the second vaccination was higher in the ChAdOx1-S/BNT vaccination group compared to the BNT/BNT vaccination group (respectively, 84 (3) days vs. 27 (6) days, *p* = 0.008). The total time duration between serology testing and prime vaccination was higher among the ChAdOx1-S/BNT vaccination group compared to the BNT/BNT vaccination group (respectively, 120 (8) days vs. 70 (10) days, *p* < 0.001) (Figure 1A).

In multivariate analysis, antibody levels for BNT/BNT remained significantly higher compared to ChAdOx1-S/BNT after adjustment for the time duration between the second vaccination and serology test (*p* < 0.001) and after adjustment for age (*p* = 0.007), but not after adjustment for the time duration between the prime and second vaccinations (*p* = 0.181) (Table 1).

In each vaccination group, no significant differences were observed for antibody levels between males and females (males vs. females for ChAdOx1-S/BNT, *p* = 0.943, and males vs. females for BNT/BNT, *p* = 0.505). However, antibodies in the BNT/BNT group were higher than for ChAdOx1-S/BNT in females (*p* = 0.001) but not in males (*p* = 0.097) (Figure 1B).

**Figure 2:**
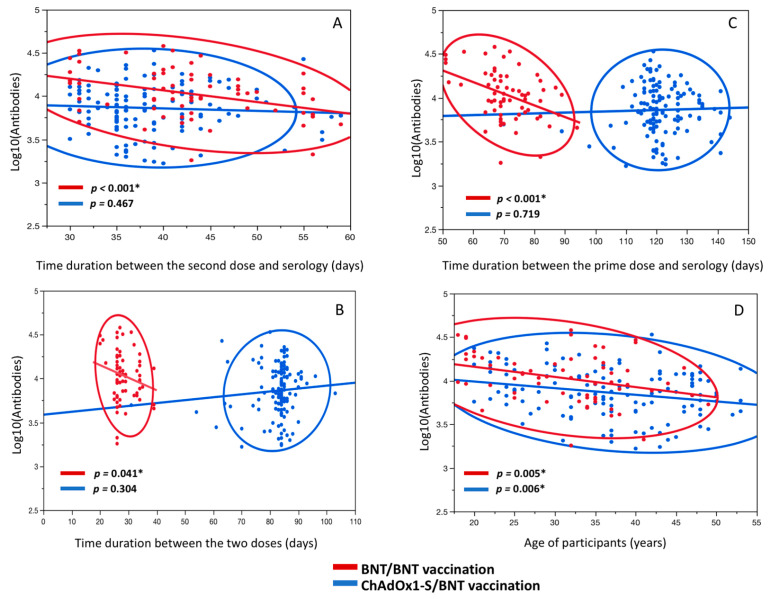
Association between IgG antibody levels and time duration aspects and age. (**A**) Association between IgG antibody levels and time duration between second vaccination and serology test for BNT/BNT and ChAdOx1-S/BNT. (**B**) Association between IgG antibody levels and time duration between prime and second vaccinations for BNT/BNT and ChAdOx1-S/BNT. (**C**) Association between IgG antibody levels and time duration between prime vaccination and serology test for BNT/BNT and ChAdOx1-S/BNT. (**D**) Association between IgG antibody levels and age of healthcare workers for BNT/BNT and ChAdOx1-S/BNT. * Significant models after adjustment for all covariates.

A negative relationship between antibody levels and time duration between the second dose and the serology test was observed for the antibody levels of BNT/BNT (*p* < 0.001), which remained significant after adjustment for all covariates (*p* < 0.001), but not for ChAdOx1-S/BNT (*p* = 0.467) (Figure 2A).

No significant correlation was observed between antibody levels and time duration between the prime and second dose for ChAdOx1-S/BNT (*p* = 0.304). No significant correlation was observed for the BNT/BNT (*p* = 0.089), but it became significant after adjustment for all covariates (*p* = 0.041) (Figure 2B). When considering the total time duration between the prime vaccination and the serology test with antibody levels, a negative correlation was observed for BNT/BNT (*p* < 0.001), which remained significant after adjustment for all covariates (*p* = 0.001), but not for ChAdOx1-S/BNT (*p* = 0.719) (Figure 2C). A negative relationship was observed for antibody levels and age for the ChAdOx1-S/BNT group (*p* = 0.007), which remained significant after adjustment for all covariates (*p* = 0.006) and for BNT/BNT (*p* = 0.008) which remained significant after adjustment for all covariates (*p* = 0.005) (Figure 2D).

## 4. Discussion

Our study showed a significant difference for antibody levels between BNT/BNT and ChAdOx1-S/BNT; however, this became non-significant after adjustment for the time duration between the prime and second doses of vaccination (Figure 1A). The change in vaccine strategy was associated with a prolonged duration between the prime and second doses for the ChAdOx1-S group, leading to a lower level of antibodies after the second dose (Figure 2C).

Recent phase 1/2 studies have shown robust immunogenicity of homologous BNT and ChAdOx1-S immunizations [20,21]. By contrast, immunogenicity of heterologous ChAdOx1-S/BNT immunization has been rarely reported [10,13,14] with preliminary results. Our results appear to be consistent with this literature, showing no significant difference in concentrations of antibodies between heterologous and homologous vaccination.

In contrast to other studies that determined the time duration between the prime and second vaccinations, we report the real-life duration after a change in vaccine strategy for people in France. Very few studies have focused on this topic, and thus the comparison of our results appears difficult. Nevertheless, previous studies have shown that immunogenicity was impacted by the time between the doses. These studies showed that the longer the interval between the prime and the second vaccination of ChAdOx1-S, the higher is the IgG spike protein-specific response [10,14].

Our results showed that there was no difference between homologous and heterologous vaccination schedules. Previous studies have suggested that cellular responses are maintained regardless of age and gender after two-vaccination schedules with homologous ChAdOx1-S and with heterologous ChAdOx1-S/BNT [10,14]. Studies reported time between first and second vaccinations as 28 days in a Com-COV study [13] and 71 days for a German study focused on healthcare workers [14], showing a similar rate of immune responses. In our study this time was different for the two groups of vaccination, with 27 days for the BNT/BNT group and 84 days for ChAdOx1-S/BNT. These delays were the clinical recommendations for these vaccines in France. Thus, we can question the clinical relevance of having retained the homologous schedule for the second vaccination in the case of applying a heterologous vaccination. In the case of heterologous vaccination, it would be more effective for the second dose to use the schedule corresponding to the additional vaccine used (i.e., BNT with three months), rather than to respect the schedule of homologous vaccination (i.e., ChAdOx1-S with three weeks).

Here, we hypothesize that the lengthening of the vaccine interval between first dose with ChAdOx1-S and second dose with BNT could be associated with a low rate of immunogenicity. The change in the French vaccine campaign strategy may be associated with a lower rate of immune response for people who received a heterologous vaccine (ChAdOx1-S/BNT) due to a delay between the prime and second vaccination.

However, our results show a significant decrease in antibodies between the second vaccination and the serology test for the homologous BNT/BNT vaccine but not for the heterologous ChAdOx1-S/BNT vaccine (Figure 2A). Thus, a possible decrease may be observed between these two vaccine strategies. It could be of interest to extend the clinical study of these healthcare workers to compare the evolution of antibodies in future prospective studies. To our knowledge, no other study has observed this result and the comparison with the literature remains difficult.

### Limitations

Our study presents potential limitations, as it is not a randomized controlled trial. Due to the current recommendations for heterologous ChAdOx1-S/BNT vaccination in people under 55 years, we could not recruit a matched cohort of homologous ChAdOx1-S/ChAdOx1-S vaccinated healthcare workers, since most of the healthcare workers have chosen the recommended heterologous booster. The majority of our healthcare workers were female, and this proportion could affect the interpretation of the results focused on gender differences. Hence, we could not define the exact action of the heterologous BNT booster vaccine compared to ChAdOx1-S homologous boosting alone. In our study, we compared the immunogenicity of homologous BNT/BNT and heterologous ChAdOx1-S/BNT vaccination. In addition to the different combinations of prime and boost vaccines, the time between first and second vaccines was significantly different in the homologous (27 days) and heterologous (84 days) groups (Table 1). Moreover, the short duration of the study after the second vaccination (i.e., 30 to 60 days) could be a limitation for interpretation of the results and future study with a longer duration of follow-up after the second vaccination should be performed to compare with our actual results. No SARS-CoV-2 anti-Spike (or anti-NC) antibody levels were collected before inclusion of participants due to the French legislation. In our study, we cannot exclude bias from participants who had previous asymptomatic COVID-19 infection, which could influence the data. No antibody serum was collected after the first dose in our study, and we cannot clearly conclude that differences in the antibody levels observed can be attributed to the different time durations between the first dose and the serology test for homologous and heterologous vaccination.

## 5. Conclusions

In conclusion, our study observed that heterologous and homologous schedules with ChAdOx1-S and BNT vaccinations present robust immune responses 30 days to 60 days after the second dose. Moreover, the results observed were equivalent after adjustment for covariates and emphasize the importance of flexibility in deploying mRNA and viral vectored vaccines. Nevertheless, applying the ChAdOx1-S vaccination schedule for the second vaccination when the BNT vaccine was administered did not seem appropriate in light of a decrease in IgG antibody levels in the heterologous vaccination compared to the homologous vaccination. The second vaccination with BNT after a ChAdOx1-S prime may be more efficient with a schedule strategy of BNT rather than with a ChAdOx1-S vaccination schedule. However, these results should be confirmed by applying prospective clinical trials.

## Figures and Tables

**Table 1:** Characteristics of the study population.

	ChAdOx1-S/BNT Vaccination		BNT/BNT Vaccination		*p* Value	*p* Value **
	*n* = 130		*n* = 67			
Age	37	(13)	32	(11)	<0.001	
Gender (Female)	104	80.0%	59	88.1%	0.156	
T1	38	(7)	42	(9)	<0.001	
T2	84	(3)	27	(6)	0.008	
T1 + T2	120	(8)	70	(10)	<0.001	
GM Antibodies *	7268.6	(6501.3–8128.3)	10,734.9	(9141.1–12,589.3)	<0.001	0.181

T1: time between second vaccination and serology test. T2: time between prime and second vaccination. T1 + T2: time between prime vaccination and serology test. * GM: geometric mean (mean with 95% confidence interval). ** *p* value for antibody levels after adjustment for T2 (time between prime and second vaccination). IQR: interquartile range. Age, T1, T2, T1 + T2 are expressed in median + (IQR). Gender is expressed in number and percentage.

## Data Availability

Not applicable.
